# Association between pre-delivery maternal red blood cell distribution width and birth weight discordance in twin pregnancies: a twin pregnancy cohort study

**DOI:** 10.3389/fpubh.2026.1912722

**Published:** 2026-10-06

**Authors:** Xiaoni Zhang, Jie Su, Li Yue, Hongyan Wang, Zhengqiang Bai, Huijie Wang, Bin Yi

**Affiliations:** 1 Center for Early Childhood Development, Gansu Provincial Maternity and Child-Care Hospital (Gansu Provincial Central Hospital), Lanzhou, China; 2 Clinical Trial Center, Gansu Provincial Maternity and Child-Care Hospital (Gansu Provincial Central Hospital), Lanzhou, China; 3 Department of Endoscopy, Shijiazhuang Traditional Chinese Medicine Hospital, Shijiazhuang, China; 4 Neonatal Intensive Care Center, Gansu Provincial Maternity and Child-Care Hospital (Gansu Provincial Central Hospital), Lanzhou, China

**Keywords:** birth weight discordance, non-linear association, red cell distribution width, twin growth discordance, twin pregnancy

## Abstract

**Background:**

Twin growth discordance, commonly defined as an inter-twin birth weight difference ≥20%, is linked to increased neonatal morbidity, but simple maternal laboratory markers to aid late-pregnancy risk assessment are limited. Red blood cell distribution width (RDW), a routine hematological parameter associated with inflammation and placental dysfunction, has been associated with adverse perinatal outcomes in singleton pregnancies; however, its role in twin growth discordance remains unclear.

**Objective:**

This study aimed to examine the association between pre-delivery RDW measured within 1 week before delivery and twin growth discordance in a cohort of Chinese twin pregnancies.

**Study design:**

This retrospective cohort study included 1,714 women with twin pregnancies who delivered at the Gansu Provincial Maternity and Childcare Hospital between July 2020 and February 2024. Pre-delivery RDW was obtained from the last prenatal complete blood count within 1 week before delivery. Twin growth discordance was defined as inter-twin birth weight discordance ≥20%. Associations were assessed using multivariable logistic regression, with RDW modeled continuously and categorically (<14.3, 14.3–16.0%, and ≥16.1%). Restricted cubic spline, subgroup, and sensitivity analyses were also performed.

**Results:**

Overall, 231 women (13.5%) had twin growth discordance. Compared with the lowest RDW category, the highest category was associated with increased odds of twin growth discordance after adjustment for maternal age, IVF status, chorionicity, gestational age, pregestational body mass index, parity, hypertensive disorders of pregnancy, gestational diabetes mellitus, and hemoglobin [odds ratio (OR) 1.89, 95% confidence interval (CI) 1.28–2.80; *p* = 0.001]. A significant trend was observed across RDW categories (*p* for trend = 0.001). RDW modeled as a continuous variable showed a borderline association after full adjustment (OR 1.06, 95% CI 1.00–1.12; *p* = 0.060). Restricted cubic spline analysis suggested a nonlinear association (*p* for non-linearity = 0.013). Results were similar using discordance ≥25% (OR 2.24, 95% CI 1.31–3.83; *p* = 0.003).

**Conclusion:**

Higher pre-delivery RDW was associated with greater odds of twin growth discordance. Prospective studies with serial RDW measurements are needed to confirm these findings and determine their clinical relevance.

## 1 Introduction

Twin growth discordance is a clinically important complication in twin pregnancies. In research and clinical practice, an intertwin birth weight difference of at least 20% is commonly used as the threshold for defining discordance (1). Approximately 16% of twin pregnancies exhibit birth weight discordance of at least 20% (2), which is associated with a higher risk of neonatal complications and other adverse perinatal outcomes (3). Previous studies have shown that multiple placental and maternal factors influence twin growth discordance (3–5). In recent years, maternal blood-based biomarkers have also been explored for prenatal assessment (6); however, the available evidence has focused mainly on specialized serum markers, whereas the potential value of routine hematological parameters has not been sufficiently studied.

Red blood cell distribution width (RDW), a standard component of complete blood count, reflects variability in erythrocyte size (7, 8). Beyond its traditional hematological use, RDW has been increasingly linked to inflammation, oxidative stress, and various adverse clinical outcomes (9–11). Pregnancy-related studies have shown that RDW is associated with several obstetric complications, with relatively consistent evidence in preeclampsia. Indeed, systematic reviews suggest that RDW levels are generally higher in women with preeclampsia than in those with normal pregnancies (12). In addition, recent studies have indicated that late pregnancy RDW is associated with several perinatal outcomes, including small or large size for gestational age, and preterm birth (13). These findings suggest that RDW may, at least in part, reflect the maternal physiological status relevant to pregnancy health and fetal growth.

Although maternal blood-based biomarkers are increasingly being explored for prenatal assessment of twin pregnancies, direct evidence regarding the relationship between pre-delivery RDW measured within 1 week before delivery levels and twin growth discordance remains limited, particularly in the Chinese population. Moreover, existing studies on RDW and pregnancy outcomes have focused primarily on singleton pregnancies or general obstetric outcomes, and its relevance to specific phenotypes of discordant twin growth remains unclear. Therefore, using data from Gansu Provincial Maternity and Childcare Hospital, this study investigated the association between pre-delivery RDW measured within 1 week before delivery levels and twin growth discordance and further explored the potential nonlinear dose–response relationship and possible threshold effect.

## 2 Materials and methods

### 2.1 Study population

This retrospective cohort study was conducted at the Gansu Provincial Maternity and Child-Care Hospital, China, and included women with twin pregnancies who delivered between July 2020 and February 2024. The study protocol was approved by the Institutional Ethics Committee of Gansu Provincial Maternity and Child-Care Hospital (No. 2022(24)). The requirement for informed consent was waived by the Ethics Committee because the study was based on retrospectively collected and de-identified clinical data, with no direct participant contact and no additional interventions. The study was conducted in accordance with the Declaration of Helsinki and is reported in accordance with the Strengthening the Reporting of Observational Studies in Epidemiology (STROBE) statement (Supplementary Table 1). The completed STROBE checklist is provided in the Supplementary materials.

Women were eligible if they had a twin pregnancy with available birth weight data for both neonates and pre-delivery RDW measured within 1 week before delivery. Exposure was defined as the pre-delivery RDW measured from the last prenatal complete blood count obtained within 1 week before delivery. The exclusion criteria were as follows: (1) termination of pregnancy before 24 weeks of gestation, (2) intrauterine death of one fetus during pregnancy, and (3) missing RDW data. After applying these criteria, 1,714 women with twin pregnancies were included in the final analysis.

### 2.2 Outcome definition

The primary outcome was intertwin birth weight discordance of at least 20% (14). Birth weight discordance was calculated as follows: birth weight discordance (%) = (larger twin’s actual weight – smaller twin’s actual weight)/(larger twin’s actual weight) × 100 (15). Twin growth discordance was defined as birth weight discordance ≥20%, consistent with commonly used definitions in twin pregnancy research (5).

### 2.3 Exposure assessment

The exposure of interest was pre-delivery RDW measured within 1 week before delivery, a routinely reported parameter in complete blood count that reflects heterogeneity in red blood cell size. In the primary analysis, RDW was analyzed both as a continuous variable (per 1% increment) and categorical variable. For categorical analyses, RDW was grouped into tertiles: T1 (<14.3%), T2 (14.3–16.0%), and T3 (≥16.1%).

### 2.4 Covariates

Potential confounders were selected based on clinical relevance and prior evidence (16) and included maternal age at delivery, *in vitro* fertilization (IVF) status, chorionicity of twins, gestational age at delivery, pregestational body mass index (BMI), parity, hypertensive disorders of pregnancy, and gestational diabetes mellitus, HGB (Hemoglobin). Maternal age was categorized as <35 and ≥35 years (17). Chorionicity was determined based on ultrasound findings. No monochorionic monoamniotic (MCMA) pregnancies were present among the included participants; thus, chorionicity was described and analyzed as dichorionic diamniotic (DCDA) or monochorionic diamniotic (MCDA). Gestational age was categorized as <37 and ≥37 weeks for the subgroup analyses. Hypertensive disorders of pregnancy were divided into three groups: those without hypertension, those with preeclampsia, and those with eclampsia.

Prior to imputation, missing data were observed only for pregestational BMI (21/1,714, 1.2%). Missing BMI values were imputed using multiple imputation by chained equations, with five imputed datasets generated. The imputation model included twin growth discordance, pre-delivery RDW measured within 1 week before delivery, and all covariates included in the multivariable models: maternal age at delivery, IVF status, chorionicity, gestational age at delivery, pregestational BMI, parity, hypertensive disorders of pregnancy, gestational diabetes mellitus, and hemoglobin. Estimates were pooled using Rubin’s rules. Participants with missing RDW values were excluded because RDW was the primary exposure variable. Given that missingness was limited to BMI and accounted for only 1.2% of the study population, formal convergence diagnostics were not performed.

### 2.5 Statistical analysis

Baseline characteristics were summarized across the RDW categories. Continuous variables were described using the mean (standard deviation) or median (interquartile range), and categorical variables were expressed as counts (percentages). Between-group comparisons were conducted using analysis of variance, the Kruskal–Wallis test, or the chi-square test, as appropriate.

Associations between pre-delivery RDW measured within 1 week before delivery and twin growth discordance were evaluated using logistic regression with effect estimates reported as odds ratios (ORs) and 95% confidence intervals (CIs). RDW was modeled both continuously (per 1% increase) and categorically (<14.3%, 14.3–16.0%, and ≥16.1%). Three sequentially adjusted models were fitted: Model 1, adjusted for maternal age, IVF status, chorionicity, and gestational age; Model 2, additionally adjusted for pregestational BMI and parity; Model 3, further adjusted for hypertensive disorders of pregnancy and gestational diabetes mellitus; and Model 4, further adjusted for HGB. Tests for trends across the RDW categories were performed by entering the median value of each category as a continuous variable.

Restricted cubic spline regression was used to examine the shape of the association between pre-delivery RDW measured within 1 week before delivery and twin growth discordance, with three knots placed at the 25th, 50th, and 75th percentiles of RDW. Threshold effects were further explored using multivariate piecewise logistic regression. Likelihood ratio testing was used to compare the piecewise and single-line models.

Prespecified subgroup analyses were conducted according to maternal age, IVF status, chorionicity, gestational age (<37 vs. ≥37 weeks), hypertensive disorders of pregnancy, and gestational diabetes mellitus. Potential effect modification across subgroups was assessed using interaction tests. Given the limited event numbers in some subgroups, these analyses were considered exploratory.

Several sensitivity analyses were performed to evaluate the robustness of the findings. First, logistic regression analyses were repeated using twin growth discordance ≥25% as an alternative outcome definition. Second, the primary analysis for twin growth discordance ≥20% was repeated in the complete-case dataset. Third, the multivariable regression models were refitted after excluding gestational age, while retaining all other covariates included in the primary analysis. Finally, to account for potential nonlinearity and the influence of extreme BMI values, the analyses were repeated after categorizing pregestational BMI according to the WHO adult BMI classification.

All analyses were performed using R software (version 4.2.2; R Foundation for Statistical Computing, Vienna, Austria) and the Free Statistics platform (version 2.4; Beijing, China). All tests were two-sided, and *p* < 0.05 was considered statistically significant.

## 3 Results

### 3.1 Study population and baseline characteristics

A total of 1,714 women with twin pregnancies were included in the final analysis, of whom 231 (13.5%) had twin growth discordance ≥20% (Figure 1). Participants were categorized into three RDW groups according to RDW levels: <14.3% (*n* = 529), 14.3–16.0% (*n* = 595), and ≥16.1% (*n* = 590). The baseline characteristics of the study population are shown in Table 1. Several maternal and pregnancy-related characteristics differed significantly across RDW categories, including maternal age, IVF status, gestational age at delivery, pregestational BMI, parity, hypertensive disorders of pregnancy, gestational diabetes mellitus, and HGB levels (all *p* < 0.05). Compared with women in the lower RDW categories, those in the highest RDW category had a lower proportion of IVF conception, lower HGB levels, and a higher prevalence of hypertensive disorders of pregnancy. The rate of twin growth discordance ≥20% also increased gradually with higher RDW categories, from 10.8% in the lowest group to 16.9% in the highest group (*p* = 0.007).

**Figure 1:**
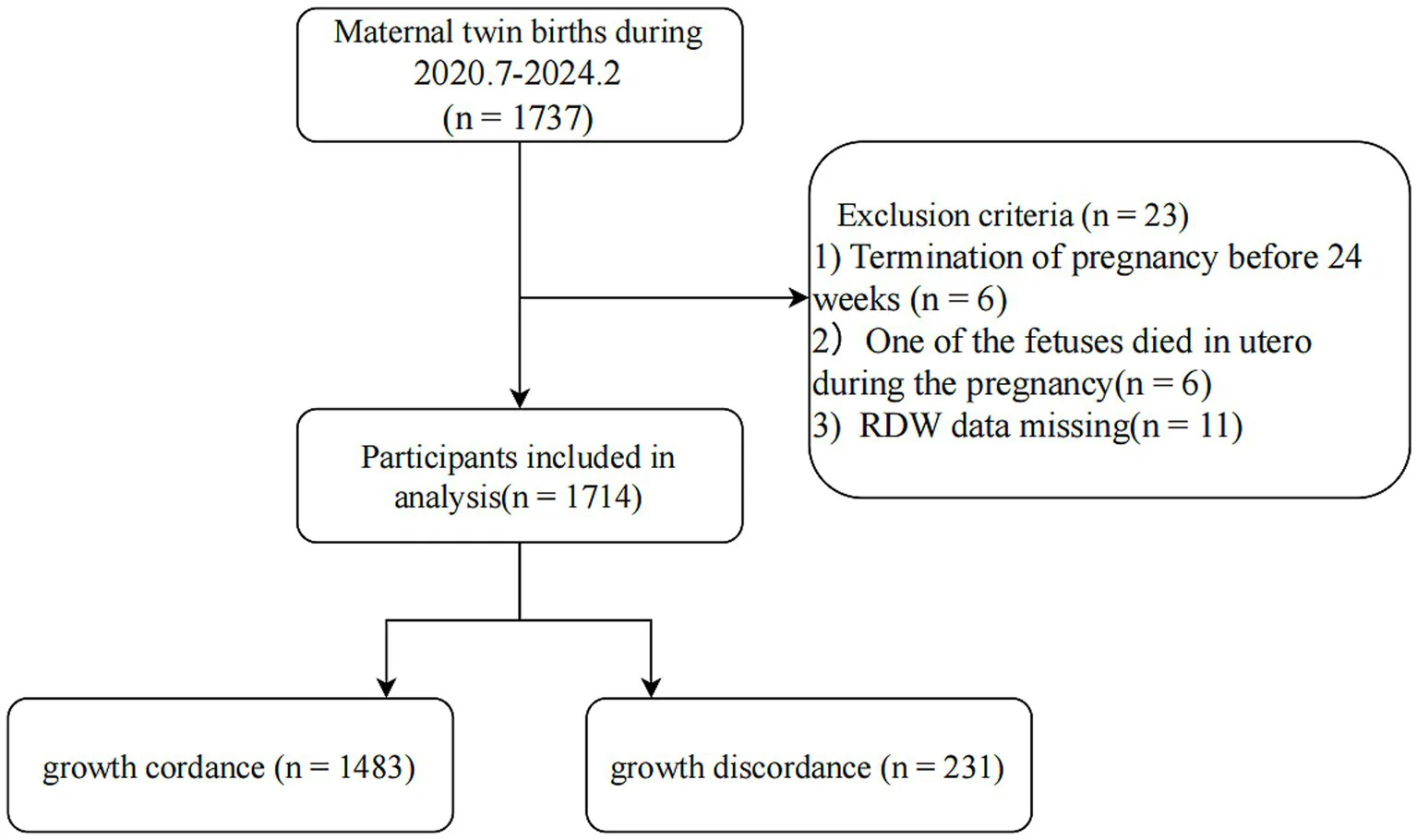
Flowchart of participant selection.

**Table 1:** Baseline characteristics of the study population according to pre-delivery maternal RDW categories.

Variables	RDW categories, %				*p-*value
	Total	T1 (<14.3)	T2 (14.3–16.0)	T3 (≥16.1)	
No.	1714	529	595	590	
Maternal age, years	30.6 ± 4.1	30.6 ± 4.0	31.0 ± 4.1	30.1 ± 4.2	0.002
Maternal age, *n* (%)					0.276
<35	1,441 (84.1)	445 (84.1)	490 (82.4)	506 (85.8)	
≥35	273 (15.9)	84 (15.9)	105 (17.6)	84 (14.2)	
IVF, *n* (%)					<0.001
No	866 (50.5)	238 (45)	281 (47.2)	347 (58.8)	
Yes	848 (49.5)	291 (55)	314 (52.8)	243 (41.2)	
Chorionicity of twins, *n* (%)					0.280
MCDA	331 (19.3)	114 (21.6)	107 (18)	110 (18.6)	
DCDA	1,383 (80.7)	415 (78.4)	488 (82)	480 (81.4)	
GA, weeks	35.7 ± 1.9	35.5 ± 2.2	35.7 ± 2.0	35.9 ± 1.5	0.009
GA, *n* (%)					0.285
<37	1,211 (70.7)	376 (71.1)	407 (68.4)	428 (72.5)	
≥37	503 (29.3)	153 (28.9)	188 (31.6)	162 (27.5)	
Pregestational BMI, (kg/m^2^)	22.0 ± 3.0	21.9 ± 3.0	22.3 ± 3.3	21.8 ± 2.8	0.003
Parity, *n* (%)					0.010
1	825 (48.1)	282 (53.3)	282 (47.4)	261 (44.2)	
2	499 (29.1)	146 (27.6)	181 (30.4)	172 (29.2)	
3	246 (14.4)	56 (10.6)	91 (15.3)	99 (16.8)	
≥4	144 (8.4)	45 (8.5)	41 (6.9)	58 (9.8)	
Hypertensive disorders of pregnancy, *n* (%)					0.008
No	1,361 (79.4)	444 (83.9)	468 (78.7)	449 (76.1)	
Preeclampsia	218 (12.7)	48 (9.1)	86 (14.5)	84 (14.2)	
Eclampsia	135 (7.9)	37 (7.0)	41 (6.9)	57 (9.7)	
Gestational diabetes mellitus, *n* (%)					0.002
No	1,479 (86.3)	459 (86.8)	491 (82.5)	529 (89.7)	
Yes	235 (13.7)	70 (13.2)	104 (17.5)	61 (10.3)	
HGB, (g/L)	117.9 ± 16.6	125.7 ± 14.7	119.5 ± 14.5	109.2 ± 16.1	<0.001
Growth discordance, *n* (%)					0.007
No	1,483 (86.5)	472 (89.2)	521 (87.6)	490 (83.1)	
Yes	231 (13.5)	57 (10.8)	74 (12.4)	100 (16.9)	

RDW, red cell distribution width; IVF, *in vitro* fertilization; MCDA, monochorionic diamniotic; DCDA, dichorionic diamniotic; GA, gestational Age; BMI, body mass index; HGB, hemoglobin; T, tertiles. Data are presented as mean ± standard deviation or *n* (%), as appropriate.

### 3.2 Association between RDW and twin growth discordance

In logistic regression analyses for twin growth discordance ≥20%, higher RDW categories were associated with increased odds of discordance. Compared with the reference group (<14.3%), the highest RDW category (≥16.1%) was associated with higher odds of discordance in the crude model (OR 1.69, 95% CI 1.19–2.40; *p* = 0.003). This association remained significant after adjustment for maternal age, IVF status, chorionicity, and gestational age (Model 1: OR 1.71, 95% CI 1.20–2.43; *p* = 0.003), additional adjustment for pregestational BMI and parity (Model 2: OR 1.72, 95% CI 1.21–2.45; *p* = 0.003), further adjustment for hypertensive disorders of pregnancy and gestational diabetes mellitus (Model 3: OR 1.63, 95% CI 1.14–2.33; *p* = 0.008), and additional adjustment for hemoglobin (Model 4: OR 1.89, 95% CI 1.28–2.8; *p* = 0.001). A significant positive trend across RDW categories was observed in all models, including the fully adjusted model (*p* for trend = 0.001). The middle RDW category was not significantly associated with discordance in any model. RDW as a continuous variable showed only a borderline association after adjustment for hemoglobin (Model 4: OR 1.06, 95% CI 1.00–1.12; *p* = 0.060) (Table 2).

**Table 2:** Logistic regression analysis of the association between pre-delivery maternal RDW and twin weight discordance ≥20% using interpolation data.

Variable	Total, n	Event, *n* (%)	Crude model		Model 1		Model 2		Model 3		Model 4	
			OR (95% CI)	*p-*value	OR (95% CI)	*p-*value	OR (95% CI)	*p-*value	OR (95% CI)	*p*-value	OR (95% CI)	*p-*value
RDW (%)	1714	231 (13.5)	1.05 (1.00 ~ 1.10)	0.067	1.05 (1.00 ~ 1.10)	0.070	1.04 (0.99 ~ 1.10)	0.085	1.04 (0.99 ~ 1.09)	0.147	1.06 (1.00 ~ 1.12)	0.060
RDW (%) tertiles												
<14.3	529	57 (10.8)	1 (Ref)		1 (Ref)		1 (Ref)		1 (Ref)		1 (Ref)	
14.3–16.0	595	74 (12.4)	1.18 (0.81 ~ 1.70)	0.386	1.18 (0.82 ~ 1.71)	0.368	1.23 (0.85 ~ 1.77)	0.281	1.18 (0.81 ~ 1.72)	0.381	1.24 (0.85 ~ 1.82)	0.255
≥16.1	590	100 (16.9)	1.69 (1.19 ~ 2.40)	0.003	1.71 (1.20 ~ 2.43)	0.003	1.72 (1.21 ~ 2.45)	0.003	1.63 (1.14 ~ 2.33)	0.008	1.89 (1.28 ~ 2.80)	0.001
*p* for trend				0.002		0.002		0.002		0.006		0.001

Model 1 adjusted for maternal age, IVF status, chorionicity, and gestational age. Model 2 additionally adjusted for pregestational BMI and parity. Model 3 additionally adjusted for hypertensive disorders of pregnancy and gestational diabetes mellitus. Model 4 additionally adjusted for HGB. OR, odds ratio; CI, confidence interval; RDW, red blood cell distribution width; IVF, in vitro fertilization; BMI, body mass index; HGB, Hemoglobin.

### 3.3 Dose–response analysis

Restricted cubic spline analysis showed a significant nonlinear association between pre-delivery RDW measured within 1 week before delivery and twin growth discordance (*p* for overall association = 0.011; *p* for non-linearity = 0.013). Using 15.1% as the reference value, the spline curve suggested that the odds of twin growth discordance increased across the lower-to-middle RDW range and then tended to attenuate at higher RDW levels, indicating a nonlinear rather than strictly linear relationship (Figure 2).

**Figure 2:**
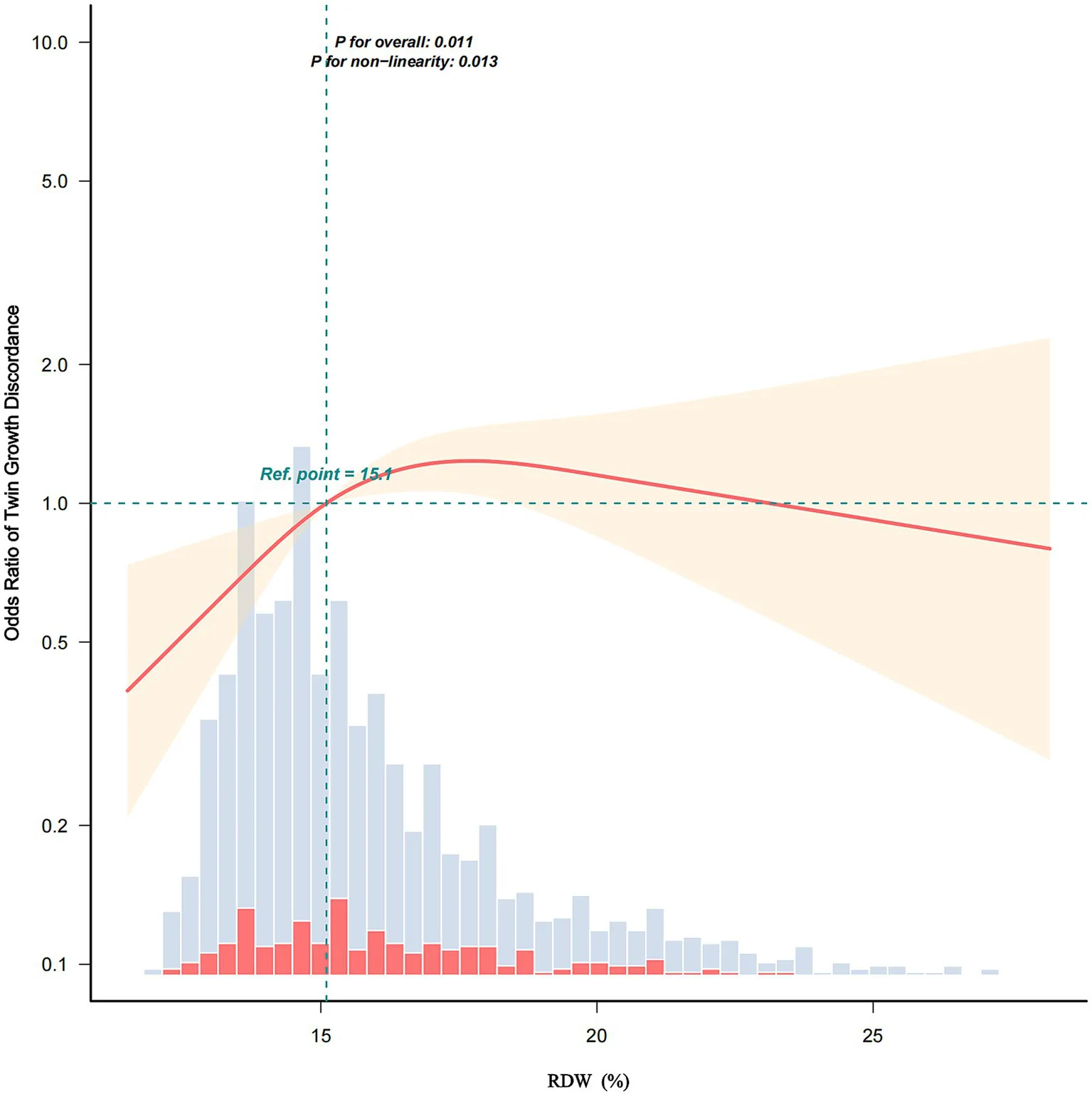
Restricted cubic spline analysis of the association between pre-delivery maternal RDW and twin growth discordance. The spline model was adjusted for maternal age, IVF status, chorionicity, gestational age, pregestational BMI, parity, hypertension during pregnancy, and gestational diabetes mellitus, Hemoglobin. Knots were placed at the 25th, 50th, and 75th percentiles of RDW. 100% of the data is shown. Solid and shaded areas represent the estimated odds ratios and 95% confidence intervals, respectively. RDW, red blood cell distribution width; OR, odds ratio; CI, confidence interval.

To further characterize this nonlinear pattern, threshold effect analysis identified a potential turning point at RDW = 18.0% (likelihood ratio, *p* = 0.001) (Table 3). Among the 1,714 participants, 1,416 had RDW values <18.0%, with 182 (12.85%) discordance events, whereas 298 had RDW values ≥18.0%, with 49 (16.44%) discordance events. Below the threshold, each 1% increase in RDW was associated with higher odds of twin growth discordance (adj. OR: 1.165, 95% CI: 1.032–1.316; *p* = 0.013). In contrast, at RDW values ≥18.0%, the association was attenuated and did not reach statistical significance (adj. OR: 0.836, 95% CI: 0.697–1.002; *p* = 0.052).

**Table 3:** Threshold effect analysis of the association between pre-delivery maternal RDW and twin growth discordance.

RDW (%)	Participants, *n*	Discordance events, *n*	Adjusted OR (95% CI)	*p* value
<18.0	1,416	182 (12.85)	1.165 (1.032 ~ 1.316)	0.013
≥18.0	298	49 (16.44)	0.836 (0.697 ~ 1.002)	0.052
Likelihood Ratio test	–		–	0.001

Adjusted for maternal age, IVF status, chorionicity, gestational age, pregestational BMI, parity, hypertensive disorders of pregnancy, gestational diabetes mellitus and HGB. OR, odds ratio; CI, confidence interval; RDW, red blood cell distribution width; IVF, in vitro fertilization; BMI, body mass index; HGB, Hemoglobin. Data are presented as *n* (%).

### 3.4 Subgroup analyses

Pre-specified subgroup analyses were conducted according to maternal age, IVF status, chorionicity, gestational age at delivery, gestational diabetes mellitus, and hypertensive disorders of pregnancy. The direction of the association between pre-delivery RDW measured within 1 week before delivery and twin growth discordance was broadly consistent across subgroups, and no statistically significant interactions were detected all (*p* for interaction > 0.05) (Figure 3). The number of events was limited, resulting in wide confidence intervals in some strata.

**Figure 3:**
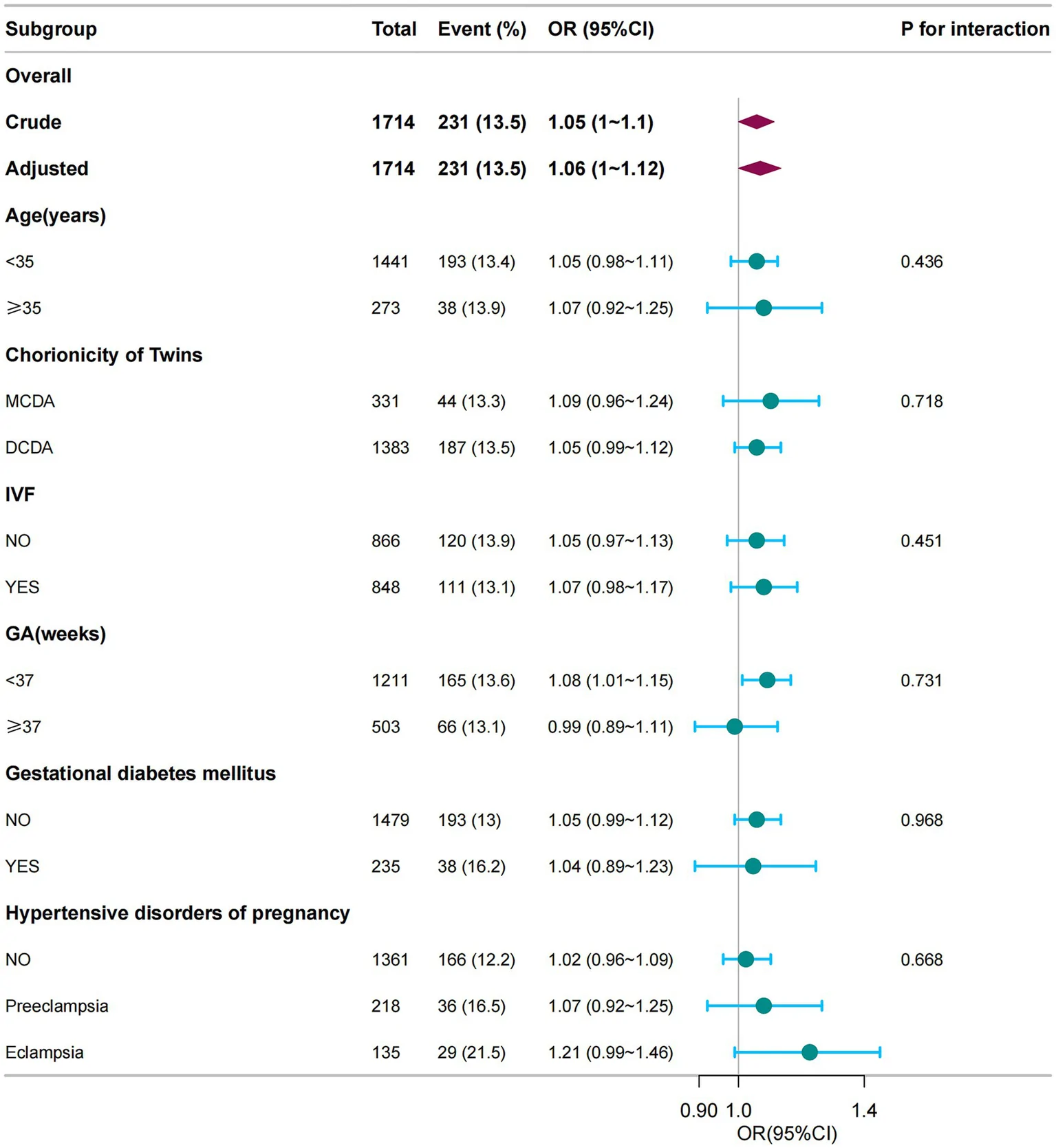
Subgroup analyses of the association between pre-delivery maternal RDW and twin growth discordance. Forest plot shows subgroup-specific adjusted odds ratios and 95% confidence intervals for the association between maternal RDW and twin growth discordance. Models were adjusted according to Model 4, except for the stratification variable in each subgroup analysis. OR, odds ratio; CI, confidence interval; IVF, *in vitro* fertilization; MCDA, monochorionic diamniotic; DCDA, dichorionic diamniotic; GA, gestational age.

### 3.5 Sensitivity analyses

When twin growth discordance ≥25% was used as an alternative outcome definition, the overall pattern remained similar (Table 4). The overall pattern was consistent with the primary analysis. In the fully adjusted model, the highest RDW category (≥16.1%) was associated with increased odds of discordance compared with the reference group (<14.3%) (OR 2.24, 95% CI 1.31–3.83; *p* = 0.003), and the trend across categories remained significant (*p* for trend = 0.003). As in the primary analysis, continuous RDW was not significantly associated with discordance after full adjustment (OR 1.04, 95% CI 0.97–1.12; *p* = 0.248), and the middle RDW category remained non-significant.

**Table 4:** Logistic regression analysis of the association between pre-delivery maternal RDW and twin weight discordance ≥25% using interpolation data.

Variable	Total, n	Event, *n* (%)	Crude model		Model 1		Model 2		Model 3		Model 4	
			OR (95% CI)	*p-*value	OR (95% CI)	*p*-value	OR (95% CI)	*p-*value	OR (95% CI)	*P*-value	OR (95% CI)	*p-*value
RDW (%)	1714	121 (7.1)	1.04 (0.97 ~ 1.11)	0.265	1.04 (0.97 ~ 1.11)	0.293	1.03 (0.97 ~ 1.10)	0.335	1.02 (0.95 ~ 1.09)	0.533	1.04 (0.97 ~ 1.12)	0.248
RDW (%) tertiles												
<14.3	529	26 (4.9)	1 (Ref)		1 (Ref)		1 (Ref)		1 (Ref)		1 (Ref)	
14.3–16.0	595	42 (7.1)	1.47 (0.89 ~ 2.43)	0.134	1.53 (0.92 ~ 2.55)	0.098	1.56 (0.94 ~ 2.60)	0.085	1.51 (0.9 ~ 2.51)	0.116	1.63 (0.97 ~ 2.74)	0.063
≥16.1	590	53 (9.0)	1.91 (1.18 ~ 3.10)	0.009	1.95 (1.19 ~ 3.20)	0.008	1.95 (1.19 ~ 3.20)	0.008	1.81 (1.10 ~ 2.98)	0.020	2.24 (1.31 ~ 3.83)	0.003
*p* for trend				0.008		0.008		0.008		0.021		0.003

Model 1 adjusted for maternal age, IVF status, chorionicity, and gestational age. Model 2 additionally adjusted for pregestational BMI and parity. Model 3 additionally adjusted for hypertensive disorders of pregnancy and gestational diabetes mellitus. Model 4 additionally adjusted for HGB. OR, odds ratio; CI, confidence interval; RDW, red blood cell distribution width; IVF, *in vitro* fertilization; BMI, body mass index; HGB, hemoglobin.

In the complete-case analysis for twin growth discordance ≥20%, the association was also robust (Table 5). The highest RDW category remained associated with increased odds of discordance in the fully adjusted model including hemoglobin (OR 1.77, 95% CI 1.20–2.62; *p* = 0.004), and the trend across categories remained significant (*p* for trend = 0.004).

**Table 5:** Logistic regression analysis of the association between pre-delivery maternal RDW and twin weight discordance ≥20% in the complete data.

Variable	Total, n	Event, *n* (%)	Crude model		Model 1		Model 2		Model 3		Model 4	
			OR (95% CI)	*p-*value	OR (95% CI)	*p*-value	OR (95% CI)	*p-*value	OR (95% CI)	*p*-value	OR (95% CI)	*p*-value
RDW (%)	1,693	227 (13.4)	1.05 (1.00 ~ 1.10)	0.074	1.05 (1.00 ~ 1.10)	0.066	1.05 (0.99 ~ 1.10)	0.083	1.04 (0.99 ~ 1.09)	0.140	1.05 (0.99 ~ 1.11)	0.093
RDW (%) tertiles												
<14.3	524	57 (10.9)	1 (Ref)		1 (Ref)		1 (Ref)		1 (Ref)		1 (Ref)	
14.3–16.0	587	73 (12.4)	1.16 (0.81 ~ 1.68)	0.420	1.17 (0.81 ~ 1.70)	0.400	1.21 (0.84 ~ 1.76)	0.312	1.17 (0.80 ~ 1.7)	0.411	1.22 (0.83 ~ 1.78)	0.305
≥16.1	582	97 (16.7)	1.64 (1.15 ~ 2.33)	0.006	1.67 (1.17 ~ 2.38)	0.005	1.68 (1.18 ~ 2.4)	0.004	1.59 (1.11 ~ 2.28)	0.011	1.77 (1.20 ~ 2.62)	0.004
*p* for trend				0.005		0.004		0.004		0.010		0.004

Model 1 adjusted for maternal age, IVF status, chorionicity, and gestational age. Model 2 additionally adjusted for pregestational BMI and parity. Model 3 additionally adjusted for hypertensive disorders of pregnancy and gestational diabetes mellitus. Model 4 additionally adjusted for HGB. OR, odds ratio; CI, confidence interval; RDW, red blood cell distribution width; IVF, *in vitro* fertilization; BMI, body mass index; HGB, hemoglobin.

In an additional sensitivity analysis excluding gestational age from the models, the highest RDW category remained significantly associated with discordance Model4 (OR 1.81, 95% CI 1.23–2.67; *p* = 0.003), with a significant trend across RDW categories (*p* for trend = 0.002) (Table 6).

**Table 6:** Logistic regression analysis of the association between pre-delivery maternal RDW and twin weight discordance ≥20% using interpolation data.

Variable	Total, n	Event, *n* (%)	Crude model		Model 1		Model 2		Model 3		Model 4	
			OR (95% CI)	*p-*value	OR (95% CI)	*p*-value	OR (95% CI)	*p-*value	OR (95% CI)	*p*-value	OR (95% CI)	*p-*value
RDW (%)	1714	231 (13.5)	1.05 (1.00 ~ 1.10)	0.067	1.05 (1.00 ~ 1.10)	0.078	1.04 (0.99 ~ 1.10)	0.095	1.04 (0.99 ~ 1.09)	0.156	1.05 (0.99 ~ 1.11)	0.094
RDW (%) tertiles												
<14.3	529	57 (10.8)	1 (Ref)		1 (Ref)		1 (Ref)		1 (Ref)		1 (Ref)	
14.3–16.0	595	74 (12.4)	1.18 (0.81 ~ 1.70)	0.386	1.18 (0.81 ~ 1.70)	0.389	1.22 (0.84 ~ 1.76)	0.300	1.18 (0.81 ~ 1.71)	0.394	1.23 (0.84 ~ 1.79)	0.285
≥16.1	590	100 (16.9)	1.69 (1.19 ~ 2.40)	0.003	1.68 (1.18 ~ 2.40)	0.004	1.7 (1.19 ~ 2.42)	0.003	1.62 (1.13 ~ 2.31)	0.008	1.81 (1.23 ~ 2.67)	0.003
*p* for trend				0.002		0.003		0.003		0.007		0.002

Model 1 adjusted for maternal age, IVF status, chorionicity. Model 2 additionally adjusted for pregestational BMI and parity. Model 3 additionally adjusted for hypertensive disorders of pregnancy and gestational diabetes mellitus. Model 4 additionally adjusted for HGB. OR, odds ratio; CI, confidence interval; RDW, red blood cell distribution width; IVF, *in vitro* fertilization; BMI, body mass index; HGB, hemoglobin.

## 4 Discussion

### 4.1 Principal findings

In this cohort of twin pregnancies, higher pre-delivery RDW measured within 1 week before delivery was associated with increased odds of intertwin birth weight discordance. After comprehensive multivariable adjustment, women in the highest RDW category (≥16.1%) exhibited significantly increased odds of twin growth discordance (≥20%) compared with those in the lowest RDW group (<14.3%), with a significant dose–response trend across graded RDW strata. Of note, continuous RDW failed to demonstrate a significant linear association with discordance, whereas restricted cubic spline analyses confirmed a nonlinear relationship between pre-delivery RDW and twin growth discrepancy. Exploratory threshold analysis identified an estimated turning point at 18.0%. Given the limited number of participants and events above this value, this turning point should not be interpreted as a validated clinical cutoff. Sensitivity analyses using a stricter discordance definition (≥25%), complete-case data, and models excluding gestational age at delivery yielded broadly consistent findings, and prespecified subgroup analyses showed no significant effect modification. Collectively, these findings indicate that higher pre-delivery RDW measured within 1 week before delivery levels are independently correlated with increased likelihood of discordant twin growth.

### 4.2 Results in the context of what is known

To date, few studies have specifically investigated the association between RDW and twin growth discordance. Current prenatal assessment strategies for twin discordance primarily rely on first-trimester ultrasound parameters and maternal serum biomarkers, including early pregnancy indicators and mid-trimester placental growth factor (18–20), while little attention has been paid to routine hematological markers such as RDW. As a classic hematological indicator widely used in obstetric research, RDW is closely implicated in pregnancy complications. Previous systematic reviews and meta-analyses have demonstrated that RDW levels are significantly elevated in women with preeclampsia compared with normal pregnancies (21, 22). Accumulating evidence further supports that late-gestation RDW is associated with multiple adverse perinatal outcomes, including small-for-gestational age, large-for-gestational age, and preterm birth (23–27). Building on these findings, the present study focused on twin pregnancies and explored the relationship between routine pre-delivery RDW measured within 1 week before delivery and twin-specific growth discordance, providing preliminary evidence for this association. Nevertheless, the generalizability of these results to broader twin populations requires further validation.

### 4.3 Clinical implications

The present findings provide valuable supplementary evidence for late-pregnancy risk assessment in twin pregnancies. Pathophysiologically, elevated late-gestation RDW may reflect an adverse maternal-placental microenvironment that contributes to discordant twin growth. Beyond reflecting erythrocyte size heterogeneity, increased RDW is a well-recognized marker of systemic inflammation, oxidative stress, and impaired erythropoiesis (9, 21, 28, 29). In pregnant women, higher RDW is strongly correlated with placenta-mediated disorders characterized by excessive oxidative stress and placental dysfunction, particularly preeclampsia (30, 31). Twin growth discordance is predominantly attributed to unequal placental territory sharing, aberrant umbilical cord insertion, and asymmetric oxygen and nutrient perfusion between the two fetuses (32, 33). Therefore, the observed association between elevated RDW and twin growth discordance may be partially explained by shared underlying placental pathophysiological abnormalities. However, this observational study cannot establish causality or determine whether RDW provides incremental predictive value beyond established clinical and ultrasound-based assessments.

### 4.4 Research implications

The RDW is a routinely available and low-cost hematological parameter. However, because RDW was measured within 1 week before delivery in this single-center retrospective study, the present findings should be regarded as hypothesis-generating rather than evidence supporting clinical implementation. Future prospective multicenter studies with serial RDW measurements should evaluate the temporal association between RDW and twin growth discordance, assess its incremental predictive value beyond established clinical and ultrasound-based indicators, and determine whether RDW has utility in risk prediction or clinical decision-making.

### 4.5 Strengths and limitations

This study has several strengths. It focused specifically on twin pregnancies, a population for which evidence on the association between pre-delivery RDW and twin growth discordance remains limited. RDW was derived from routine prenatal complete blood count testing and is therefore readily available. In addition, we applied complementary analytical approaches, including multivariable regression, trend analysis, restricted cubic spline modeling, exploratory subgroup analyses, and sensitivity analyses using alternative outcome definitions and complete-case data, and models excluding gestational age at delivery from the covariate adjustment set to assess the robustness of the observed associations.

Several limitations merit consideration. First, owing to the retrospective observational design, causal inference is not possible. Second, selection bias and residual confounding cannot be completely excluded, despite multivariable adjustment, subgroup analyses, and sensitivity analyses. In particular, information on maternal iron or folate supplementation and detailed anemia diagnoses was unavailable in this retrospective dataset. Moreover, although RDW has been linked to inflammation, elevated RDW may represent a nonspecific marker or proxy for underlying placental-mediated disorders and associated maternal conditions, rather than a direct driver of twin growth discordance. Third, RDW was measured within 1 week before delivery; therefore, it should be interpreted as a pre-delivery associated marker rather than an early screening or predictive marker. Finally, this was a single-center study, which may limit the generalizability of the findings. Accordingly, multicenter prospective studies with serial RDW measurements are needed to confirm the observed association and further clarify its clinical relevance.

## 5 Conclusion

In conclusion, higher pre-delivery RDW measured within 1 week before delivery was associated with increased odds of twin birth weight discordance, with evidence of a nonlinear relationship. Prospective multicenter studies with repeated RDW measurements are needed to validate these results and determine their clinical relevance.

## Data Availability

The original contributions presented in the study are included in the article/Supplementary material, further inquiries can be directed to the corresponding author.
